# An Effective Algorithm to Analyze the Optokinetic Nystagmus Waveforms from a Low-Cost Eye Tracker

**DOI:** 10.3390/healthcare10071281

**Published:** 2022-07-10

**Authors:** Wei-Yen Hsu, Ya-Wen Cheng, Chong-Bin Tsai

**Affiliations:** 1 Department of Information Management, National Chung Cheng University, Chiayi 621, Taiwan; shenswy@mis.ccu.edu.tw (W.-Y.H.); u09556003@alum.ccu.edu.tw (Y.-W.C.); 2 Center for Innovative Research on Aging Society, National Chung Cheng University, Chiayi 621, Taiwan; 3 Advanced Institute of Manufacturing with High-Tech Innovations, National Chung Cheng University, Chiayi 621, Taiwan; 4 Department of Ophthalmology, Ditmanson Medical Foundation Chiayi Christian Hospital, Chiayi 600, Taiwan; 5 Department of Optometry, College of Medical and Health Science, Asia University, Chiayi 600, Taiwan

**Keywords:** low-cost eye tracker, optokinetic nystagmus, waveform analysis

## Abstract

Objective: Most neurological diseases are usually accompanied by changes in the oculomotor nerve. Analysis of different types of eye movements will help provide important information in ophthalmology, neurology, and psychology. At present, many scholars use optokinetic nystagmus (OKN) to study the physiological phenomenon of eye movement. OKN is an involuntary eye movement induced by a large moving surrounding visual field. It consists of a slow pursuing eye movement, called “slow phase” (SP), and a fast re-fixating saccade eye movement, called “fast phase” (FP). Non-invasive video-oculography has been used increasingly in eye movement research. However, research-grade eye trackers are often expensive and less accessible to most researchers. Using a low-cost eye tracker to quantitatively measure OKN eye movement will facilitate the general application of eye movement research. Methods & Results: We design an analytical algorithm to quantitatively measure OKN eye movements on a low-cost eye tracker. Using simple conditional filtering, accurate FP positions can be obtained quickly. The high-precision FP recognition rate is of great help for the subsequent calculation of eye movement analysis parameters, such as mean slow phase velocity (MSPV), which is beneficial as a reference index for patients with strabismus and other eye diseases. Conclusions: Experimental results indicate that the proposed method achieves faster and better results than other approaches, and can provide an effective algorithm to calculate and analyze the FP position of OKN waveforms.

## 1. Introduction

Eye movement related research has brought considerable contributions to various fields such as visual and detection science, psychology and neurobiology [1,2,3]. Therefore, robust non-invasive eye detection and gaze tracking are critical for developing human-computer interaction, neurological disease diagnosis, and understanding human emotional states. Electrooculography, scleral search coil systems, and video eye diagrams are all common methods of measuring eye movement. In recent years, these methods have been used more and more frequently in video-ophthalmology due to their non-invasive advantages [4,5]. Optokinetic reflex, which is driven by the optokinetic system, consists of the slow involuntary eye movements induced by a large, moving, visual field. It is a basic mechanism to stabilize the image of the outside world on the retina in a moving environment. If the visual field keeps moving continuously, optokinetic nystagmus (OKN) will occur. OKN is composed of slow pursuing eye movements, “slow phase” (SP), and the resetting saccades, “fast phase” (FP). The production of OKN depends on the integrity of several neural pathways, including retinal photoreceptors, afferent pathway of retinal ganglion cells, lateral geniculate body, occipital lobe, cerebellar flocculus, paramedian pontine reticular formation, and the efferent pathway of the ocular motor neurons. Any dysfunction of these pathways may lead to change in the OKN.

Video-oculography has been used increasingly in eye movement research because of its advantages of non-invasiveness. There have been several models of high-sampling rate, high-resolution, research-grade eye trackers for current eye movement research. These models are often expensive and operate in closed-source software, which limits their clinical application.

In the current research literature, several methods for gaze estimation have been developed, which can generally be divided into two categories: appearance-based methods and feature-based methods. Appearance-based methods often depend on the quality and diversity of training data, and the generalization ability of regression algorithms. Methods using low-resolution images under different environmental conditions [6,7,8,9] are proposed, which address the gaze estimation problem by learning a mapping function directly from eye images to gaze directions. The difficulty faced by these methods is that the appearance of the eyes depends not only on the shape of the subject’s own eyes, but also on the angle and direction of gaze, variability in head posture, imaging conditions, etc., so they are usually not accurate enough in clinical trials. Feature-based methods extract local features (such as contours, eye corners, and eye reflections) and are widely used in gaze estimation. These methods derive eye features from high-resolution eye images by magnifying the subject’s eye. There are two main problems with these methods. The first is that since the mapping function is different for each subject and system configuration, a tedious calibration process must be performed before each test to obtain the necessary parameters. The second problem is that, once the eye positioning calibration is performed, the subject’s head must remain stationary, otherwise there will be considerable errors between the actual orientation and the system’s estimated orientation.

Efficient eye localization is an important key to obtain accurate gaze estimates. A novel complete representation (CR) pipeline with two novel eye center localization methods [10] is proposed. The first method employs geometric transformations to estimate the rotation difference between two faces (original and newly generated frontal faces) and employs an unknown localization strategy to predict CR centers. The second method is based on image translation learning, using CR-region to train a generative adversarial network (CR-GAN) and accurately generate and localize the eye center. A novel iris ripple filter [11] is proposed to improve the accuracy and robustness of gradient localization. In addition, a new depth correspondence point conversion method is also proposed, which can effectively solve the instability problem of CR-GAN in the process of eye generation and the positioning accuracy of eyeballs with subtle changes. GazeNet [12], an appearance-based depth gaze estimation method based on a 16-layer VGG deep convolutional neural network, is proposed. A new implicit calibration method [13] is introduced that exploits four natural constraints during eye gaze tracking, which helps formulate implicit calibration as a constrained unsupervised regression problem and can be solved by the hard-EM algorithm.

With the vigorous development of related research, more and more low-cost video-based eye trackers have been developed for future development of clinical applications [14]. In this study, we use a low-cost eye tracker to quantitatively measure OKN eye movement. We develop an algorithm to detect the slow and fast phases of OKN. In the process of eye movement recording, unnecessary noise generation or eye movement often occurs due to the subject’s fatigue or distraction, which affects the analysis of eye movement signals. The proposed algorithm can eliminate the interference caused by head movement and eye wandering, and optimizes the detection results of FP. In order to verify whether the performance of the proposed method meets the expected results, we compare the FP detection results with the results manually annotated by professional physicians. The experimental results show that our results are almost consistent with the results judged by these physicians.

## 2. Materials and Methods

**Figure 1:**
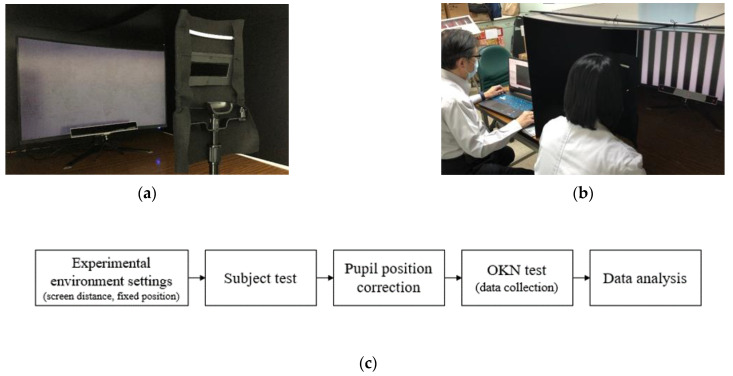
(**a**) Test environment for collecting OKN data. The eye tracker was placed beneath the screen. A chin rest was used to stabilize the subject’s head and a black facial shield was used to reduce noise signal during the test; (**b**) The screen displayed the dynamic OKN stimuli and the eye movement signal was captured by the eye tracker; (**c**) The experimental process.

We designed a series of dynamic stripe videos of OKN stimuli at different speeds, and recorded the subjects’ eye-tracking data by using a low-cost eye-tracking device in a dark and undisturbed experimental environment. In order to reduce the measurement error caused by head shaking, a chin rest was used to fix the subject’s head position during the experiment and a black facial shield was used to reduce the noise signal during the experiment. Figure 1 shows an overall overview of the experimental environment and the composition of the eye tracker. Technically explained, gaze tracking is the process of determining the point-of-gaze (POG), observing the visual axis of the subject’s computer screen or eyes in 3D space. The video ophthalmology system obtains the subject’s eye signal from an eye-tracking instrument and sends it to the personal computer for signal processing, in order to estimate the subject’s gaze direction based on the information obtained from the eye area and possible head pose.

### 2.1. Materials

Eighteen healthy adults were recruited for this study. The participants did not have any neurological or ophthalmic damage. The subjects underwent a complex test, and the measured signals collected overt OKN signatures for these 18 subjects. The study was approved by the Institutional Review Board at the Ditmanson Medical Foundation Chiayi Christian Hospital and complied according to the term of the Declaration of Helsinki [15]. All participants signed an informed consent form for study participation and were informed that they had the right to opt out.

The visual stimulus was displayed on a 32 inches curved LCD monitor (VX3218, ViewSonic, Brea, CA, USA) at a frame rate of 150 Hz. Participants were seated on a chair, with a chin rest, with their eyes at 65 cm from the monitor. The dimensions of the viewing area were 69.8 cm horizontally and 39.3 cm vertically, which equals to viewing angles of 56.5 degrees horizontally and 33.6 degrees vertically at the distance of 65 cm. The OKN responses were elicited with a square-wave grating alternative black and white vertical stripes of 3.5 cm width (equal to 3.1 degrees) moving horizontally. The participants were instructed to look at the moving patterns binocularly. In a test session, the monitor displayed moving stripes in one direction for 20 s, followed by a blank screen for 15 s, and then moving stripes in the opposite direction. The participants were asked to close their eyes during the blank screen period. The visual stimuli were displayed with the stripes moving at speeds of 10, 20, 30, and 40 degrees per second subsequently. The whole test session lasts for 5 min. Elicited eye movements were recorded using a video-based eye tracker (GP3HD, Gazepoint, Vancouver, Canada) that captured the eye movements at a sampling rate of 150 Hz. The eye movements of both eyes were captured simultaneously.

### 2.2. Proposed OKN Eye Movement Measure System

After a series of experiments and data collection, the data is preprocessed and its characteristics are determined. The original physiological signal is usually mixed with many interference factors. OKN with noise includes the failure of the machine to detect pupils, the deviation of the subject’s gaze position, etc. These may cause errors in the processing of the signal, which limits the performance of subsequent analyses, so it is necessary to filter out the noise in the signal through the steps of data preprocessing. 

**Figure 2:**
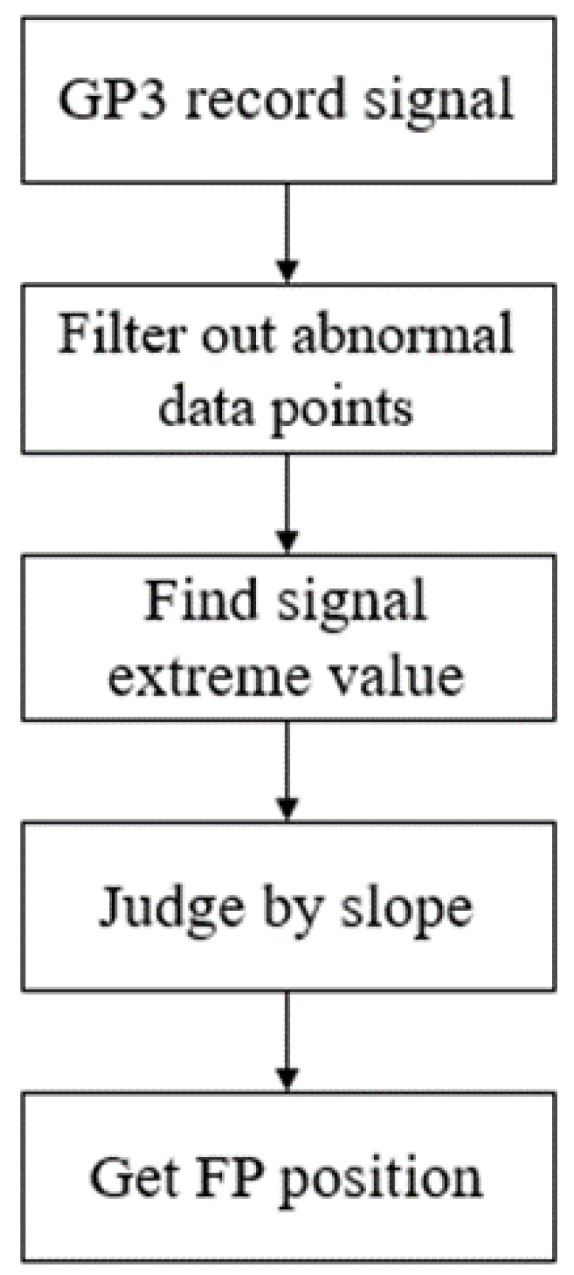
Flowchart of proposed FP position detection.

The OKN response alternates between compensatory slow phases (SP) in the direction of retinal sliding and saccadic fast phases (FP) in the opposite direction. We refer to the condition of FP generation proposed by Kei Kanari et al. (2017) [16]: the displacement slope between two points must be higher than a certain threshold. First, the collected signal is segmented and compared with its adjacent data points to find the location of the maximum and minimum values. Then, the method of judging the slope is used to correct and fine-tune the data points that meet the conditions after screening, and then the correct position of FP can be found, as shown in Figure 2.

The algorithm, Algorithm 1, is as follows (wherein the matrix is the data point of the maximum/minimum value of the signal, after connecting the points into a line, and the subsequent calculation is performed by judging the slope).
**Algorithm 1** Proposed FP position detection1for i = 1: length (X)2 find maximum/minimum of points to matrix U 3end4for i = 1: length (U)5 if (U’s slope meet M)6  find highest and lowest points of FP7 end8endX:is the input dataM:is the slope thresholdU:is the matrix after find maximum/minimum of points from input

The detailed processing steps are as follows:

#### 2.2.1. Abnormal Data Points Filtering

The causes of noise include environmental human factors and biological human factors. Environmental human factors refer to the interference caused by the environment and the operation of the equipment when the subject is measuring the signal; biological human factors refer to the physiological state of the subject, such as eye fatigue, head movement, etc., resulting in this interaction.

**Figure 3:**
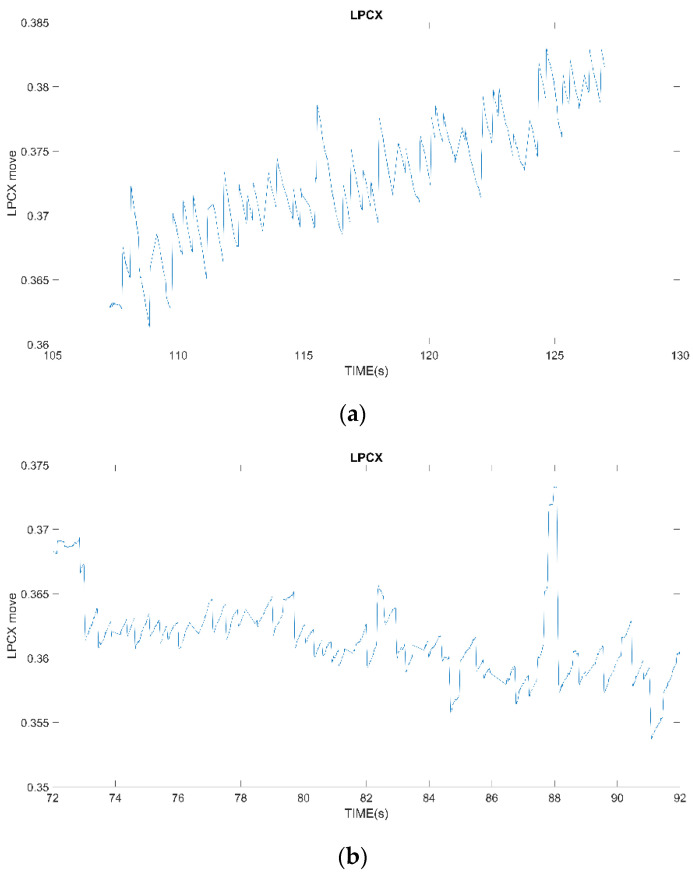
(**a**) Unable to look at the same level; the drop from the starting position is too large; (**b**) The eyeball drifts involuntarily, and a signal with excessive displacement is detected.

Before the experiment, the subjects are asked to focus on the center of the computer screen, but staring at the computer screen for a long time will still cause the subjects’ eyes to fatigue, and the eyes will drift, the reaction will become sluggish and the subjects will blink frequently. These disturbances will cause deviations in the subsequent processing of signals. Even if the collected signals have an OKN response, this may be because the subjects’ eyes are not looking at the same range, as shown in Figure 3a, or because of zoning out, looking elsewhere. The magnitude of eye movement changes greatly, as shown in Figure 3b. Various reasons make it difficult to find the highest or lowest point of the fixed frequency of the signal.

**Figure 4:**
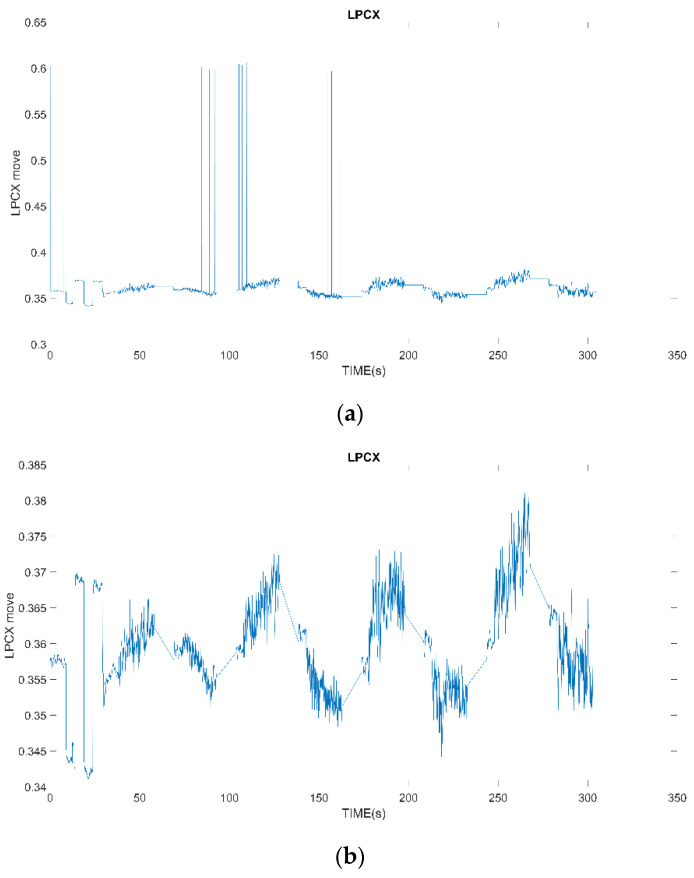
(**a**) Original data; (**b**) Noise filtered data.

The procedure of filtering out abnormal data points is listed in Algorithm 2. It was found that the extreme values of displacement in the signal are often found in data far from the initial central fixation point (computer screen). In the experimental video, the distance between the eyes is corrected by 20 degrees on the left and right sides of the computer screen width, which is equivalent to a distance of about 0.031 mm between the pupils of the two eyes. We first obtain the positions of the subjects’ eyes fixed at 20 degrees to the left and right of the computer screen width when performing binocular correction, and calculate the value of the subjects’ gaze at the center of the computer screen. The width of the computer screen is about 30 degrees on the left and right, which is equivalent to a distance of about 0.046 mm (0.031*3/2) between the pupils of the two eyes. That is to say, when the pupil displacement of the left and right eyes exceeds the central fixation point plus or minus 0.023 mm (0.046/2), it means that the subject’s eyes have looked away from the computer screen, or the measurement is disturbed. The data at this time are outliers and should be deleted and not included in the processing. An example of noise filtering is shown in Figure 4.
**Algorithm2** Proposed filter out abnormal data points1delete (LPCX > (CL + 0.023))2delete (LPCX < (CL − 0.023))3delete (RPCX > (CR + 0.023))4delete (RPCX < (CR − 0.023))LPCX:is the X-coordinates of the left eyeRPCX:is the X-coordinates of the right eyeCL:is the central fixation point of the left eyeCR:is the central fixation point of the right eye

#### 2.2.2. Signal Extrema Search

Using the signal length as the threshold, the data points in the signal are gradually compared, and the data point is compared with its neighboring data points. When a data point is larger than the previous data point, it means that the maximum value has been found. When a data point is smaller than the previous data point, it means that the minimum value is found. The locations of these data points are the places where SP and FP phenomena are speculated to occur. However, considering that there are many fluctuations in the signal, and due to factors, such as nystagmus, the movement of the eyeball may be slightly changed instantaneously, so it may not be possible to obtain an accurate extreme value. To improve the accuracy when judging extreme values, this method is designed so that the gap between two adjacent extreme values must be greater than a certain threshold (the displacement amplitude of the eyeball must be greater than 0.001 mm). That is to say, when a certain extreme value is searched, the pupil displacement amplitude of this point should be at least a certain distance from the last searched extreme value, otherwise it will not be included in the processing. This method can be used to filter the phenomenon of the signal not being smooth due to nystagmus, and more accurately predict the highest and lowest points when the FP phenomenon occurs.

#### 2.2.3. Judging by the Slope

**Figure 5:**
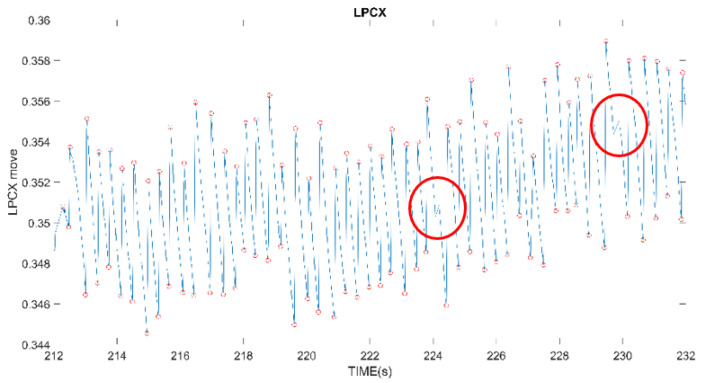
The gap (red-circled) between two adjacent extreme values is too small to be processed.

**Figure 6:**
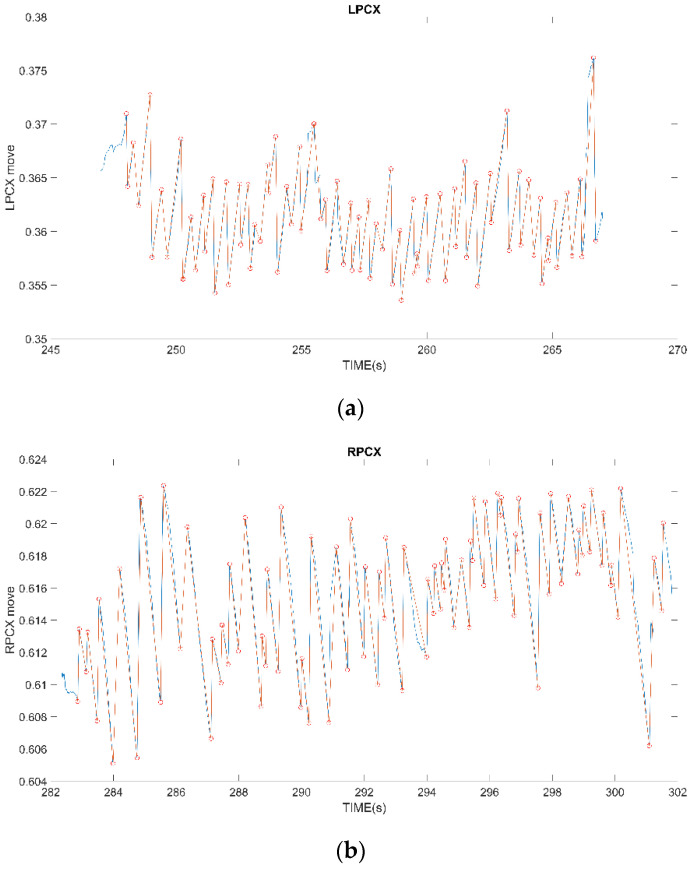
(**a**) Left-shifted striped OKN test; (**b**) Right-shifted striped OKN test.

OKN is composed of the SP generated by the eyeball following the direction of stimulus movement, and the FP that is opposite to the direction of stimulus movement. We can observe that the positions of extreme data points detected in Figure 5 all occur at the alternation of the waveform of the eye movement signal. After obtaining the data points of all extreme values, the maximum values and the minimum values are divided two by two into groups to form a line, which represents the occurrence of SP and FP phenomena, as shown in Figure 6.

In order to correctly judge FP, we further examine the slope when FP occurs, and fine adjustments and corrections are made to facilitate other subsequent OKN studies. The slope is the ratio of the difference between the pupil displacement and its time difference to indicate the degree of inclination. The function of the slope is to correctly judge and distinguish SP and FP phenomena, so as to avoid misjudgment caused by confusion. We use the slopes of the detected maxima and their adjacent minima, as well as the dynamic streak film stimulus orientation of the trial, to judge SP versus FP phenomena.

The OKN test in this experiment is a dynamic fringe film that shifts left and right at various speeds. In Figure 5, the movement trajectory of the subject’s eye gaze position in the OKN test can be observed with a left and right displacement. The OKN phenomenon connected by the extreme values has a positive slope when the stimulus movement direction is moving to the left. (The SP phenomenon is a positive slope, which means that the subject’s eye movement direction is to follow the stimulation direction to the left in the video. The negative slope here is the FP phenomenon, which means that the subject’s eyeball resets the saccade movement). Conversely, when the direction of the stimulus is moving to the right, it exhibits a negative slope.

## 3. Experimental Results and Discussion

### 3.1. Quantitative Measurement of Elicited OKN

**Figure 7:**
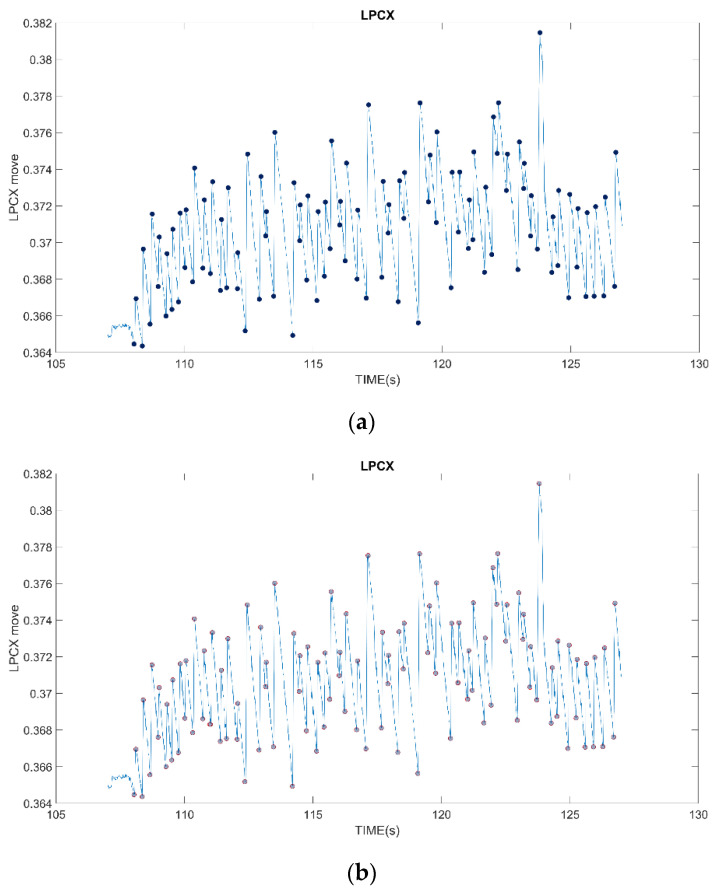
Results of proposed method. (**a**) Results marked by physicians; (**b**) The results of this method were compared with those marked by physicians.

Professional physicians were asked to assist in manually marking the correct location of FP occurrence, as shown in Figure 7a, and made an overlapping control map to compare the location of FP occurrence screened out by our proposed method with the location marked by physicians (blue dots) to verify the correctness of the proposed method. The results show that the proposed method can find out the FP occurrence position of 99% of OKNs. The reason for not full identification is that some special signals are difficult to filter, which is explained in detail in C. For normal OKN signals, 100% of the locations where FP occurs can be selected.

### 3.2. Comparisons with the State-of-the-Art Approaches

**Figure 8:**
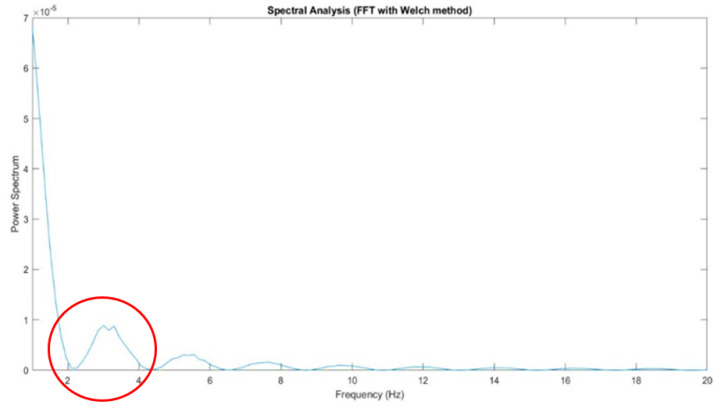
Fourier transformation with Welch method; the maximum spectral response is about 2~4 Hz.

The traditional experimental method adopts the method of Fourier transformation combined with band-pass and high-pass filtering, and uses the Welch method [17] to split the signal and perform spectrum analysis. The formula is shown in (1). The frequency at which the OKN phenomenon occurs can be observed approximately in the range of 2~4 Hz, as shown in Figure 8. The callback of this frequency control GP3HD is in line with the subject’s current saccade state, that is to say, in the experimental setting, the subject has an OKN phenomenon of about 2 to 4 times per second.

(1) $${\tilde{P}}_{PER}\left(\omega \right)=\frac{1}{MUL}\sum_{i=1}^{L}{\left|\sum_{n=0}^{M-1}x_{N}^{i}\left(n\right)d_{2}\left(n\right)e^{-j\omega m}\right|}^{2}$$

**Figure 9:**
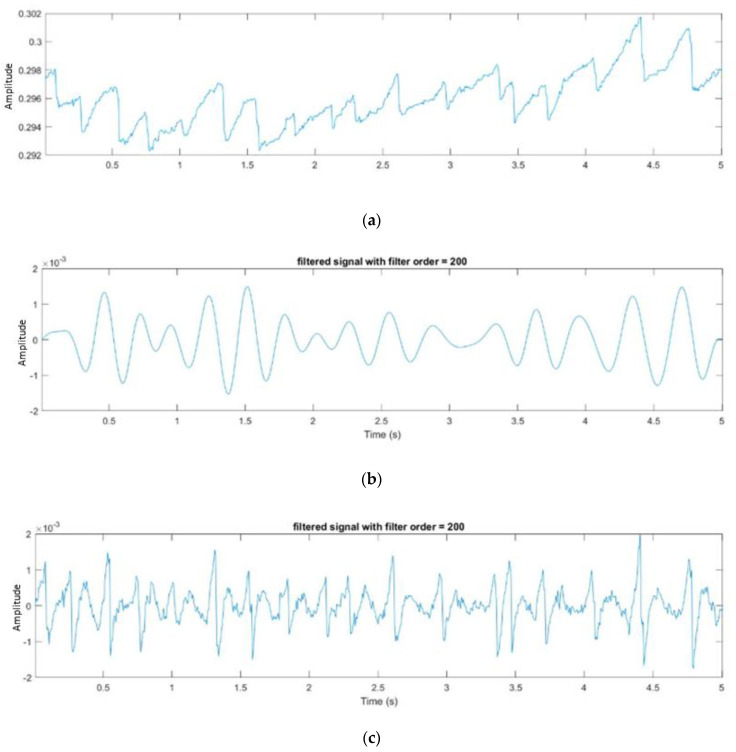
(**a**) Original signal; (**b**) Band-pass filtered signal; (**c**) High-pass filtered signal.

Fourier transformation is then used to find out the frequency of FP phenomenon; the formula is shown in (2). In the peak detection part, high-pass and band-pass FIR filtering is used first, then an adjustable threshold is set for filtering, the filtered peak is found, and the adjacent maximum and minimum values are found according to their positions. It can be observed from Figure 9 that it is easier to find the position of the FP using the results of high-pass filtering, but its disadvantage is that a threshold must be set as a condition for screening the correct point. Otherwise, some FP points may be missed.

(2) $$S\left(k\right)=\frac{x_{0}}{2}+\sum_{i=1}^{N}\left(x_{i}\cos\left(2\pi \frac{i}{T}k\right)+b_{i}\sin\left(2\pi \frac{i}{T}k\right)\right)\;\;$$

Table 1 shows the comparison between our proposed method and other methods. At first, we thought that we only needed to use Peak finding [18] to accurately find the instantaneous high point when FP occurs, but this method is very susceptible to noise interference and causes misjudgments, resulting in lower accuracy. The application of Fourier transformation and filtering [19] depends on the judgment of the set value, and high-pass filtering was selected exclusively by professional researchers as the easiest, so the accuracy rate is lower and the filtering is higher, and the FP points can be selected more correctly.

According to observation, no matter what the OKN recognition rate is, under the condition of long-term testing the subjects will generate uncontrolled noise, and these disturbances will cause deviations in the subsequent processing of the signal, thereby affecting the accuracy rate. The proposed method has already filtered out the abnormal data points in the original signal, and screened the position of FP by the slope and the magnitude of the eyeball displacement. Only the issue of dull gaze has not been completely resolved, and the FP judgment of OKN under normal conditions and the method of FFT coupled with high-pass filtering can successfully find the accurate FP point, and the result of FP judgment of OKN with noise is also the best.

### 3.3. Research Contributions and Limitations

When OKN detection is applied in clinical practice, it most often requires quite expensive instruments, and such a heavy burden makes it difficult for clinical applications to proceed smoothly. The main results are: (1) OKN detection using low-cost instruments; (2) the accurate FP position is of great help in calculating and analyzing the eye disease status of the subjects. Using simple conditional filtering, accurate FP positions can be obtained quickly.

This method can find the exact FP position without error in a stable situation where the eyeball is not largely stalled. Because of the irregular occurrence of abnormal nystagmus and sluggishness, these data samples are not included in the FP/SP reference values. Although the proposed method can currently filter out these irregular signals, the FP accuracy of the complete signal identification will still decrease due to these irregular states in the subjects.

## 4. Conclusions and Future Work

Accurate and detailed eye movement analysis will be an important reference to help understand eye diseases. The main method used in many eye movement measurement equipment is “pupil center corneal reflection” (PCCR) [22]. This method uses a near-infrared light source to illuminate the subject’s eyes, combines the bright and dark pupil effects to obtain the position of the center of the pupil, and estimates the gaze direction from the vector formed by the subtraction between the corneal reflections [23,24]. However, these methods have high requirements for equipment; in addition to cost issues, they also require complex and calibrated system settings, which are usually difficult to apply in general clinical practice.

To solve this problem, we develop an effective algorithm to recognize FP position in the OKN waveform using a low-cost eye tracking device. The algorithm can filter out irregular signals, reduce noise, and locate the position of FP correctly on a low-cost eye tracker. The experimental results indicate that our method’s results are almost consistent with the results judged by professional physicians. This proves that correct FP detection results can also be obtained using a low-cost eye tracker. Accurate FP recognition is crucial to the subsequent calculation of various OKN eye movement parameters, including mean slow-phase velocity, and the gain derived from the ratio of induced slow-phase velocity to the velocity of the stimuli. This helps us gain a deeper understanding of eye diseases and drives the development and possibilities of other related research areas.

In future work, with these important parameters of OKN eye movement, we can establish the baseline data of health subjects under different stimuli velocity and different stimuli direction. With these baseline data, the characteristics of OKN response of subjects with oculomotor disorders, including paralytic strabismus, congenital nystagmus, or acquired nystagmus, will be established. It is expected that an affordable low-cost eye tracker will become a more readily available diagnostic tool for general daily clinical practice. Applying these findings in the clinic can also benefit more patients and improve their lives.

## Figures and Tables

**Table 1:** FP recognition rate of each method in OKN and OKN with noise.

Methods	Normal OKN Signals	*p*-Value	OKN Signals with Noise	*p*-Value
Peak Finding [14]	0.43	<0.001	0.35	<0.001
FFT plus Band-Pass Filter [20]	0.96	<0.05	0.86	<0.01
FFT plus High-Pass Filter [20]	1	>0.05	0.91	<0.05
Our Previous Method [21]	1	>0.05	0.97	<0.05
Proposed Method	1	-	0.99	-

## Data Availability

Not applicable.
